# Lesão transfixante de carótida por espinha de peixe - relato de caso

**DOI:** 10.1590/1677-5449.202200121

**Published:** 2022-06-27

**Authors:** José Julio Bechir Maués, Heather Lynn Hauter Maués, Rafael Maia de Sousa, Luiz Nazareno França de Moura, Isabela Nascimento Duarte Rodrigues

**Affiliations:** 1 Hospital Jean Bitar, Belém, PA, Brasil.; 2 Hospital Ophir Loyola, Belém, PA, Brasil.

**Keywords:** fish bone, carotid lesion, esophageal perforation

## Abstract

Accidental fish bone ingestion is a common complaint at emergency departments. The majority of cases have a benign course. However, serious complications such as esophagus perforation, cervical vessel injury and cervical abscess can occur in 7.4% of cases. Mortality rates can be as high as 50% when mediastinitis occurs. We report a case of an esophageal perforation caused by a fish bone with a lesion to the right common carotid artery after 20 days of evolution. Surgical exploration occurred with corrections of the lesion in the right common carotid and esophagus. Early identification of this kind of injury is paramount to prevent potentially fatal complications.

## INTRODUÇÃO

A ingestão acidental de espinha de peixe constitui uma causa comum de atendimento em unidades de emergência. A maioria dos casos apresenta evolução benigna^1^. No entanto, complicações sérias como perfuração de esôfago, lesão de vasos cervicais e abscesso cervical podem acontecer em 7,4% dos casos^1^^-^ 3. A mortalidade pode chegar a 50% quando há evolução para mediastinite^2^^,^ 3.

A lesão da artéria carótida cervical por ingestão de corpo estranho é rara^4^ e apresenta prognóstico desfavorável se diagnosticada tardiamente devido a perda volumosa de sangue, obstrução de vias respiratórias e infecção cervical grave. Relatamos o caso de uma paciente com perfuração de esôfago e carótida comum direita decorrente de ingestão acidental de espinha de peixe.

## DESCRIÇÃO DO CASO

**Figura 1:**
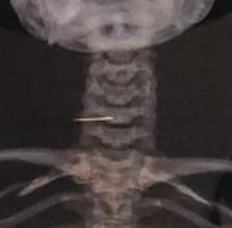
Corpo estranho paraesofágico direito.

A paciente, 27 anos, procedente de uma Unidade de Pronto Atendimento (UPA) devido ao quadro de hematêmese, febre e odinofagia iniciados há cerca de 20 dias, após ingestão acidental de espinha de peixe, referia dispneia e disfagia. Ao exame físico, apresentava-se em estado geral regular, consciente e orientada em tempo e espaço, sem icterícia, sem cianose, afebril e com palidez (2+/4+). A orofaringe não apresentava alterações, mas havia dor à palpação superficial da região cervical direita com sinais flogísticos no local. Durante a investigação do quadro de hemorragia digestiva, foi realizada uma endoscopia digestiva alta, que não evidenciou fonte de sangramento. A tomografia computadorizada sem contraste de pescoço revelou imagem de coleção mal definida paraesofágica direita com importante edema de partes moles, promovendo compressão posterior sobre o lobo direito da tireoide e o esôfago para a esquerda, que se estendia ao músculo esternocleidomastoideo direito. Havia, ainda, uma imagem de corpo estranho de aspecto alongado no interior da coleção supracitada, medindo 2,8 × 0,3 cm (Figura 1).

**Figura 2:**
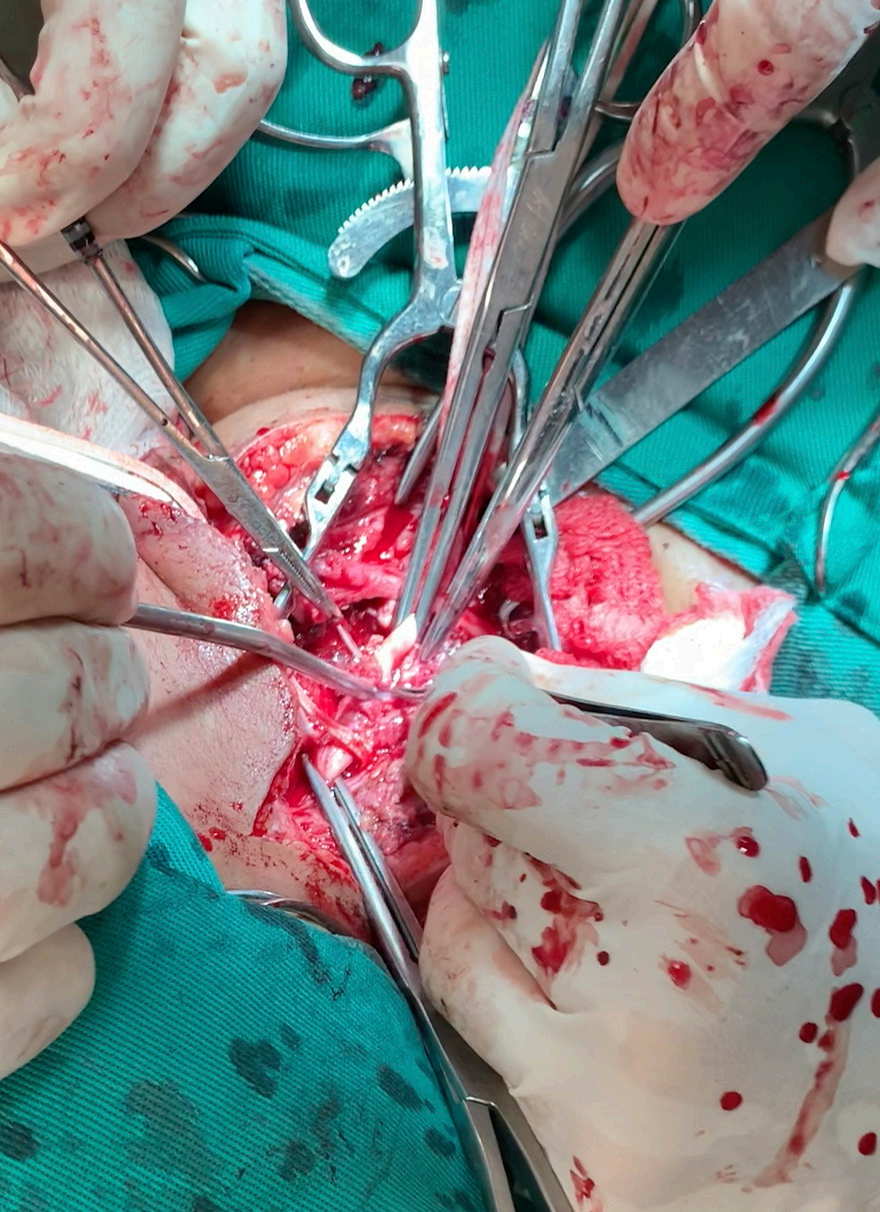
Processo inflamatório na topografia da artéria carótida direita.

**Figura 3:**
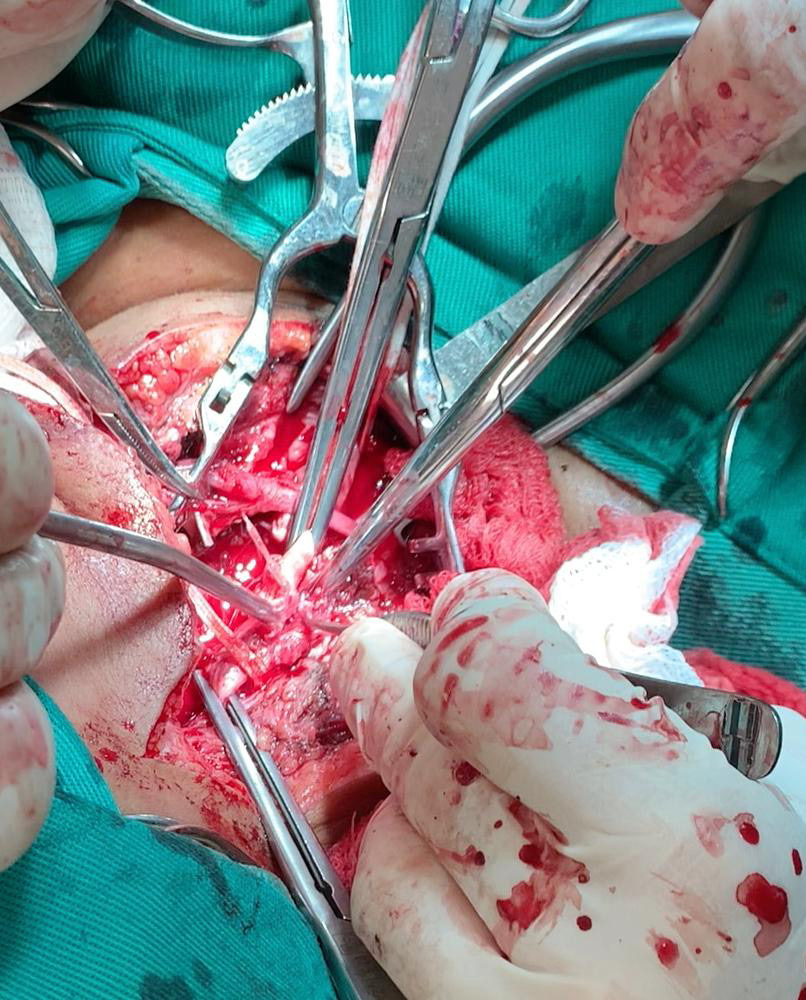
Espinha de peixe transfixando artéria carótida comum.

**Figura 4:**
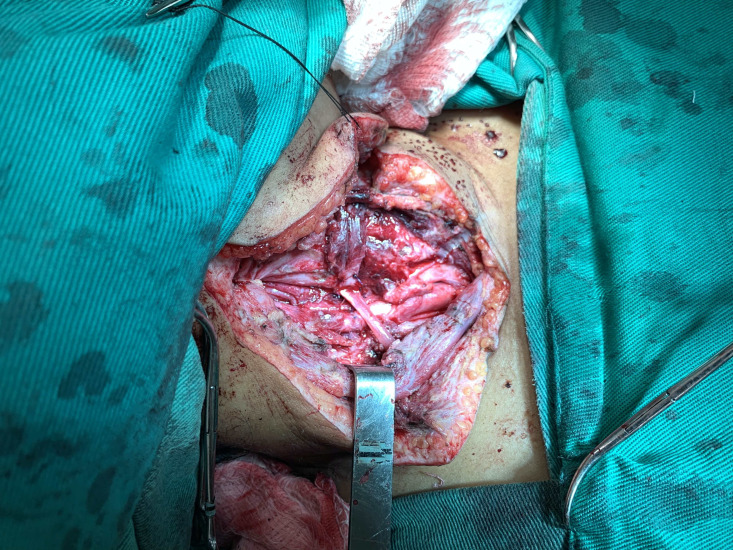
Artéria carótida comum direita após remoção da espinha de peixe, desbridamento e sutura.

Durante a internação, a paciente evoluiu com hematêmese franca, necessitando de intervenção cirúrgica de urgência, na qual foi submetida à cervicotomia exploradora direita, sendo identificado processo inflamatório extenso em região cervical direita com abscesso e sangramento ativo, em jato, em topografia de artéria carótida direita (Figura 2). Foi observada lesão transfixante da carótida comum direita por corpo estranho serrilhado, além de lesão transfixante da parede lateral direita do esôfago (Figura 3). Após heparinização sistêmica com 1 mL de heparina não fracionada, foi realizado clampeamento proximal e distal de artéria carótida comum direita, com retirada do corpo estranho, desbridamento do tecido inflamatório periarterial, seguida de sutura com prolene 6-0, pontos separados (Figura 4). Foi realizada esofagorrafia com Caprofyl 4-0, pontos separados e interposição de retalho do ventre posterior do músculo digástrico. Houve boa evolução durante a internação com progressão da dieta enteral para dieta oral liquidificada com boa tolerância. Em retorno no ambulatório de cirurgia geral cerca de 3 meses após a cirurgia, o relato foi de ingestão regular de alimentos sólidos sem nenhuma queixa. Não houve retorno no ambulatório de cirurgia vascular para realização de Doppler. As tentativas de contato para acompanhamento foram malsucedidas devido ao local de residência da paciente ser remoto.

## DISCUSSÃO

A perfuração da carótida cervical por ingestão de espinha de peixe é rara, mas pode ser catastrófica e potencialmente fatal se não for prontamente identificada^4^. Os mecanismos de lesão consistem em perfuração aguda com sangramento maciço e penetração gradual do corpo estranho na parede arterial com formação de pseudoaneurisma com posterior ruptura^4^^,^ 5. A hematêmese de pequena monta que, muitas vezes, precede um sangramento mais volumoso deve ser valorizada na investigação de lesão arterial associada^4^. Wang et al.^4^ analisaram 3.018 admissões por ingestão de corpo estranho, mostrando que a espinha de peixe é o corpo estranho mais frequentemente ingerido, e, em 3% dos casos analisados, houve lesão da artéria carótida. A hematêmese e a massa cervical pulsátil são sintomas importantes na investigação de ingestão de corpo estranho^5^. 

Os pacientes que chegam ao setor de emergência com queixa de corpo estranho devem ser examinados com visualização direita da orofaringe com auxílio de um abaixador de língua. Quando o corpo estranho não for diretamente visualizado, deve ser procurado com exames complementares antes de o paciente ser liberado^7^^,^ 8.

O uso de radiografia cervical para a identificação de espinha de peixe apresenta sensibilidade baixa^7^^,^ 8. A tomografia é o exame de escolha devido à sensibilidade >90% para detecção de espinha de peixe, além de demonstrar complicações como abscessos e lesões vasculares e possibilitar a análise do formato, tamanho e localização do corpo estranho^8^^,^ 9.

Sintomas como odinofagia, disfagia ou sensação de corpo estranho ao deglutir não conseguem determinar a localização exata do corpo estranho. A base da língua, as tonsilas, a parede posterior da faringe, o recesso ariepiglótico e o esôfago superior nos locais de estreitamento anatômico são, em ordem decrescente, as áreas mais frequentes de impactação da espinha de peixe^8^^,^ 10 ^,^ 11.

Uma vez identificado um corpo estranho alojado nos tecidos cervicais, ele deve ser prontamente removido, tendo em vista que o atraso na remoção acarreta sequelas graves^12^. Nesse caso, o cirurgião deve estar apto a realizar uma cervicotomia exploradora com dissecção delicada das estruturas cervicais em busca do corpo estranho.

No caso citado, devido à evolução de 20 dias, houve formação de abscesso cervical, com desorganização anatômica e grande quantidade de tecido inflamatório, o que tornou o procedimento mais complexo, com sangramento ativo ocorrendo durante a exploração. Dessa forma, a exploração cirúrgica é o método mais eficaz para controle de hemorragia quando há lesão vascular, além de permitir desbridamento, drenagem de abscesso e correção da perfuração esofágica.

## CONCLUSÃO

O caso relatado mostra que o atraso no diagnóstico e no tratamento de perfuração esofágica por corpo estranho pode levar a complicações graves com potencial risco de óbito. Também evidencia que relatos de dor cervical e odinofagia associadas à ingestão de peixe devem ser valorizados, e a investigação para presença de corpo estranho deve ser completa, evitando que uma lesão potencialmente grave não seja identificada.
