# Supplementary material for: Lesão transfixante de carótida por espinha de peixe - relato de caso
Source: J Vasc Bras. 2022 Jun 27;21:e20220012. [Article in Portuguese] doi: 10.1590/1677-5449.202200121 (PMC9242429; doi:10.1590/1677-5449.202200121)
Supplement: Translation

# Translation: Carotid artery perforation by fish bone - a case report

**DOI:** 10.1590/1677-5449.202200122

**Authors:** José Julio Bechir Maués, Heather Lynn Hauter Maués, Rafael Maia de Sousa, Luiz Nazareno França de Moura, Isabela Nascimento Duarte Rodrigues

**Affiliations:** 1 Hospital Jean Bitar, Belém, PA, Brasil.; 2 Hospital Ophir Loyola, Belém, PA, Brasil.

**Keywords:** fish bone, carotid lesion, esophageal perforation

## Abstract

Accidental fish bone ingestion is a common complaint at emergency departments. The majority of cases have a benign course. However, serious complications such as esophagus perforation, cervical vessel injury and cervical abscess can occur in 7.4% of cases. Mortality rates can be as high as 50% when mediastinitis occurs. We report a case of an esophageal perforation caused by a fish bone with a lesion to the right common carotid artery after 20 days of evolution. Surgical exploration occurred with corrections of the lesion in the right common carotid and esophagus. Early identification of this kind of injury is paramount to prevent potentially fatal complications.

## INTRODUCTION

Accidental fish bone ingestion is a common complaint at emergency departments. The majority of cases have a benign course.^1^ However, serious complications such as esophagus perforation, cervical vessel injury, and cervical abscess can occur in 7.4% of cases.^1^-^3^ Mortality rates can be as high as 50% when mediastinitis occurs.^2^,^3^

Lesion of the cervical carotid artery by ingestion of a foreign body is rare.^4^ It has an unfavorable prognosis if diagnosed late due to massive blood loss, airway obstruction, and severe cervical infection. We report the case of a patient with perforation of the esophagus and right common carotid artery caused by accidental fish bone ingestion.

## CASE REPORT

The patient, 27 years old, coming from a Unidade de Pronto Atendimento (UPA) due to hematemesis, fever, and odynophagia that had started 20 days prior, after accidental fish bone ingestion, reporting dyspnea and dysphagia. On physical examination, she was in regular general condition, conscious and oriented in time and space, anicteric, no cyanosis, afebrile, and pale (2+ / 4+). The oropharynx showed no alterations, superficial palpation of the cervical region resulted in pain and phlogistic signs were identified at the site. During the investigation of gastrointestinal bleeding, she underwent an upper gastrointestinal endoscopy, which showed no source of bleeding. Non-contrast computed tomography of the neck revealed a poorly defined right paraesophageal collection with significant soft tissue edema, promoting posterior compression on the right thyroid lobe and the esophagus to the left, which extended to the right sternocleidomastoid muscle. There was also an image of an elongated foreign body inside the aforementioned collection measuring 2.8 × 0.3 cm (Figure 1).

**Figure 1:** Right paraesophageal foreign body.

During hospitalization, she developed frank hematemesis, requiring urgent surgical intervention, in which she underwent an exploratory cervicotomy and an extensive inflammatory process was identified in the right cervical region with abscess and active bleeding, in the topography of the right carotid artery (Figure 2). A transfixing lesion of the right common carotid artery by a serrated foreign body was observed, in addition to a transfixing lesion of the right side of the esophagus wall (Figure 3). After systemic heparinization with 1 ml of unfractionated heparin, proximal and distal clamping of the right common carotid artery was performed with removal of the foreign body, debridement of the peri-arterial inflammatory tissue, followed by suture with 6.0 Prolene, separate stitches (Figure 4). Esophageal suture was performed with Caprofyl 4.0, separate stitches and interposition of posterior digastrics muscle belly flap. There was a good evolution during hospitalization with progression from enteral diet to liquefied oral diet with good tolerance. Upon return to the general surgery ambulatory approximately 3 months after surgery, the report was of regular solid food intake without any complaints. There was no return to the vascular surgery ambulatory clinic for Doppler imaging. Attempts to contact for follow-ups were unsuccessful due to the patient's place of residence being remote.

**Figure 2:** Inflammatory process in the topography of the right carotid artery.

**Figure 3:** Fish bone transfixing common carotid artery.

**Figure 4:** Right common carotid artery after fish bone removal, debridement, and suturing.

## DISCUSSION

Perforation of the cervical carotid by fish bone ingestion is rare, but it can be catastrophic and potentially fatal if it is not promptly identified.^4^ The lesion mechanisms consist of acute perforation with massive bleeding and gradual penetration of the foreign body into the arterial wall with formation of a pseudoaneurysm with subsequent rupture.^4^,^5^ Wang et al.^4^ analyzed 3018 admissions for ingestion of foreign body and showed that fish bone is the most frequently ingested foreign body and in 3% of the analyzed cases, there was damage to the carotid artery. Minor hematemesis that often precedes more massive bleeding should be valued in the investigation of associated arterial injury.^4^ Hematemesis and pulsatile cervical mass are important symptoms in the investigation of foreign body ingestion.^5^

Patients arriving at the emergency department with a foreign body complaint should be examined with a right view of the oropharynx with the aid of a tongue depressor. When the foreign body cannot be directly visualized, complementary exams should be sought before the patient is released.^7^,^8^

The use of cervical radiography to identify fish bones presents low sensitivity.^7^,^8^ Tomography is the exam of choice due to the sensitivity > 90% for fishbone detection, in addition to displaying complications such as abscesses and vascular lesions and making it possible to analyze the shape, size, and location of the foreign body.^8^,^9^

Symptoms such as odynophagia, dysphagia, or the sensation of a foreign body when swallowing cannot determine the exact location of the foreign body. Base of tongue, tonsils, posterior pharyngeal wall, aryepiglottic folds, and upper esophagus in locations of anatomical narrowing are, in decreasing order, the most frequent areas of fish bone impaction.^8^,^10^,^11^

Once a foreign body lodged in the cervical tissues is identified, it must be promptly removed, considering that the delay in removal leads to severe sequelae.^12^ In this case, the surgeon must be able to perform an exploratory cervicotomy with delicate dissection of the cervical structures in search of the foreign body.

In the cited case, due to the 20 day evolution, a cervical abscess was formed, with anatomical disorganization and a large amount of inflammatory tissue, which made the procedure more complex, with active bleeding occurring during exploration. Thus, surgical exploration is the most effective method for controlling bleeding when there is vascular injury, in addition to allowing debridement, abscess drainage, and correction of esophageal perforation.

## CONCLUSION

The reported case shows that delay in the diagnosis and treatment of esophageal perforation by a foreign body can lead to serious complications with a potential risk of death. It also shows that reports of cervical pain and odynophagia associated with fish ingestion must be valued and the investigation for the presence of a foreign body must be thorough, preventing a potentially serious injury from being unidentified.
